# Immune and Non-Immune Inflammatory Cells Involved in Autoimmune Fibrosis: New Discoveries

**DOI:** 10.3390/jcm12113801

**Published:** 2023-05-31

**Authors:** Margherita Sisto, Sabrina Lisi

**Affiliations:** Department of Translational Biomedicine and Neuroscience (DiBraiN), Section of Human Anatomy and Histology, University of Bari “Aldo Moro”, 70124 Bari, Italy; sabrina.lisi@uniba.it

**Keywords:** autoimmunity, inflammation, fibrosis

## Abstract

Fibrosis is an important health problem and its pathogenetic activation is still largely unknown. It can develop either spontaneously or, more frequently, as a consequence of various underlying diseases, such as chronic inflammatory autoimmune diseases. Fibrotic tissue is always characterized by mononuclear immune cells infiltration. The cytokine profile of these cells shows clear proinflammatory and profibrotic characteristics. Furthermore, the production of inflammatory mediators by non-immune cells, in response to several stimuli, can be involved in the fibrotic process. It is now established that defects in the abilities of non-immune cells to mediate immune regulation may be involved in the pathogenicity of a series of inflammatory diseases. The convergence of several, not yet well identified, factors results in the aberrant activation of non-immune cells, such as epithelial cells, endothelial cells, and fibroblasts, that, by producing pro-inflammatory molecules, exacerbate the inflammatory condition leading to the excessive and chaotic secretion of extracellular matrix proteins. However, the precise cellular mechanisms involved in this process have not yet been fully elucidated. In this review, we explore the latest discoveries on the mechanisms that initiate and perpetuate the vicious circle of abnormal communications between immune and non-immune cells, responsible for fibrotic evolution of inflammatory autoimmune diseases.

## 1. Introduction

Fibrotic autoimmune disorders are a group of chronic pathologies characterized by a damage in self-tolerance to a broad variety of autoantigens in which fibrosis develops as the end-result of a chronic inflammatory process [1]. The pathogenesis of autoimmunity involves dysfunction of the entire immune system, including neutrophils among the innate immune cells, B and T cells of the adaptive immunity, dendritic cells, and macrophages [2]. Within the various cell types related to fibrotic autoimmune diseases’ pathogeneses, non-immune cells, such as epithelial cells, endothelial cells, and fibroblasts, are considered to be key players in the occurrence and progression of these diseases [3]. Based on these premises, immune and non-immune inflammatory cells are considered to be accountable for tissue failure in a wide range of fibrotic autoimmune disorders such as rheumatoid arthritis (RA), systemic lupus erythematosus (SLE), primary Sjögren’s syndrome (pSS), and systemic sclerosis (SSc) [4]. Indeed, a plethora of recent advances has documented the functional role of inflammatory cells as therapeutic targets in autoimmune disorders [5]. However, major questions and controversies in the field remain and the comprehension of the different mechanisms that trigger fibrosis in autoimmune diseases is a challenge for many researchers. This review collects the latest advances in understanding how an alteration in the delicate balance between immune and non-immune cells is at the basis of the fibrotic evolution that is observed in various autoimmune diseases. A list of autoantigens associated with autoimmune fibrosis was reported in Table 1.

## 2. The Role of Immune and Non-Immune Inflammatory Cells in Fibrotic Autoimmune Diseases: New Discoveries

Inflammatory process is considered to be one of the main steps leading to fibrosis in autoimmune diseases [6]. Numerous studies have demonstrated that the pathophysiology of fibrosis in autoimmune diseases involves an aberrant interplay between the immune and non-immune systems [7]. Both immune and non-immune responses play an essential role in the early events of fibrosis. Dysregulation of these processes comprises inflammatory changes, including proliferation of ECM-producing cells and the occurrence of mononuclear cell inflammatory infiltrates. In this context, both immune and non-immune cells have been implicated as important active participants in inflammatory processes involving fibrotic autoimmune diseases [8]. This section will review new insights on the role of immune and non-immune inflammatory cell types in fibrotic autoimmune diseases.

### 2.1. Current Understanding of the Involvement of Immune Cells in Fibrotic Autoimmune Diseases

Both innate and adaptive immunity are involved in fibrogenesis of autoimmune diseases and, interestingly, altered orchestration of the immune system might be an early event of fibrosis [7]. Dysregulation of these processes results in autoimmune responses triggered by T lymphocytes, macrophages, or dendritic cells [2]. These activated immune cells highly secrete factors that modulate inflammatory process and rapidly promote progressive fibrosis, involving the activation of resident fibroblasts and their transformation in myofibroblasts [2,7,8]. The following paragraphs report the recent discoveries on the role of immune cells in the fibrotic evolution of autoimmune pathologies.

#### 2.1.1. Update on the Correlated Pro-Fibrotic Role of CD4+ and CD8+ T Cells

Traditionally, B lymphocytes and CD4+ T lymphocytes are considered to be key cells in the immunopathogenesis of autoimmune diseases and they have already been widely studied and are well recognized [9]. However, more recently, studies have demonstrated the increasing evidence that CD8+ T cells, infiltrating inflamed tissues, cooperate to induce tissue fibrosis in autoimmune diseases [10]. Emerging studies reported that CD8+ T cells infiltrate the lesioned skin of patients with SSc, predominantly in the early stage of the disease and exert a pro-inflammatory and pro-fibrotic activity through the induction of tissue damage [11,12]. Of particular note, key pro-fibrotic mediators, such as interleukin (IL)-6, through their signal activate CD8+ T cells and promote their interactions with fibroblasts, leading to the deposition of extracellular matrix (ECM) and contributing to the perpetuation of the fibrotic process in SSc patients [13,14]. High levels of the profibrotic type 2 cytokine IL-13 were produced following activation of peripheral blood effector CD8+ T cells from patients with SSc as compared with healthy controls or with patients with RA. In contrast, CD4+ T cells showed a lower and more variable level of IL-13 production. This abnormality was correlated with the extent of fibrosis and with a high grade of cutaneous involvement [11]. The role of CD4+ T cells is controversial because, recently, Sakkas and collaborators demonstrated that in SSc a great number of T cells of TH2 type is detected, producing pro-fibrotic IL-4, IL-13, and IL-31; in addition, CD4+ cytotoxic T lymphocytes are increased in skin lesions, and cause fibrosis and endothelial cell apoptosis [15].

**Figure 1:**
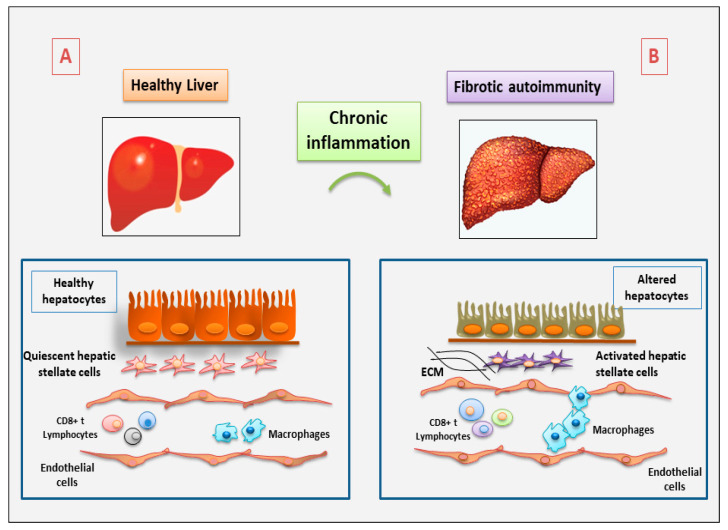
Schematic representation of the involvement of the immune cells in autoimmune hepatitis, derived from experimental mouse models (ECM = extracellular matrix) (A: Healthy liver; B: Fibrotic liver).

A key role for CD8+T cells was also demonstrated in SLE nephritis; Zhang and colleagues showed that tubule-interstitial CD8+ T cells correlate with clinic-histologic kidney impairment in SLE nephritis, determining an evident progression of interstitial fibrosis and, thus, tubular organ atrophy [16]. In addition, the expression of cytotoxic T cells is increased and the inactivation of CD4+ T cells induces fibrosis and injury of the liver tissue in patients affected by autoimmune hepatitis [17]. Autoimmune hepatitis is a progressive inflammatory liver disease characterized by chronic inflammation of the liver, circulating autoantibodies, hypergammaglobulinemia, and progressive liver fibrosis [18]. CD8+ T lymphocytes may have a significant influence on liver fibrosis and intravascular effects. After activation, CD8+ T cells usually differentiate into cytotoxic T lymphocytes, which represent effector cells that destroy tumor cells and infected cells. Actually, the function of CD8+ T lymphocytes in hepatic fibrosis needs further investigation because their role is unclear. In the liver, the activity of immune surveillance of the CD8+ T cells against virus-infected cells seems to be reduced in mice with liver fibrosis caused by HBV infection [19]. Additionally, in an experimental mice model of carbon tetrachloride-induced liver fibrosis, the transfer of splenic CD8+ T cells into the mice had the effect of exacerbating fibrosis, a process that can be prevented by IL-10 treatment [20]. On the contrary, a reduction of the number of CD8+ T cells had little effect on the progression of hepatic fibrosis in carbon tetrachloride-treated animals [21]. Given that spleen-derived CD8+ T cells induce liver fibrosis and that hepatic CD8+ T-cell depletion probably has no effect on liver fibrosis, various subtypes of CD8+ T-cell may be distributed differently in the spleen and liver of mice, playing distinct roles in liver fibrosis [22] (Figure 1).

**Figure 2:**
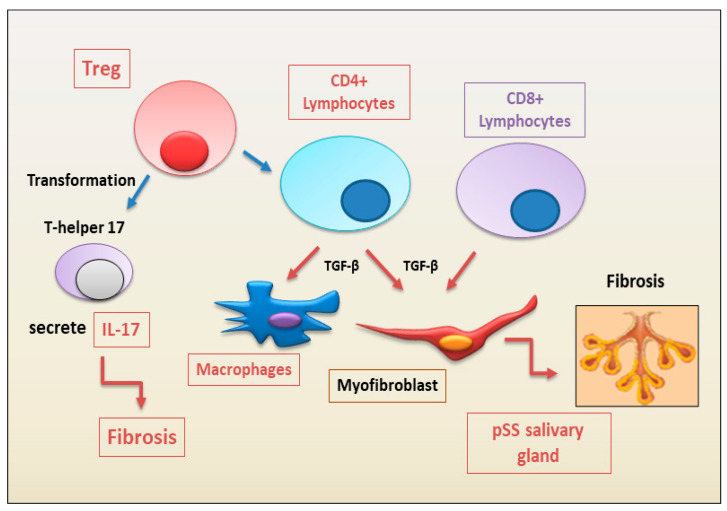
Immune cells involved in the fibrosis of salivary glands observed in the chronic inflammatory autoimmune diseases primary Sjögren’s syndrome (pSS), according to the most recent findings. The figure highlights the role of TGF-β as pro-fibrotic factor (Treg = regulatory T lymphocytes; TGF-β = tumor growth factor beta).

CD8+ T lymphocytes are also crucial players in the mechanism of exocrine gland injury in pSS [23,24]. In fact, CD8+ T lymphocytes contribute to acinar injury in the salivary glands, triggering a worsening fibrotic event in pSS [12,23]. Joachims et al. [25] showed that expanded clones of memory CD4+ T cells in the salivary glands displayed sequence similarity both within expanded clones of the same individual and among different patients, indicating that these cells are able to recognize shared antigens. They also observed that an increased frequency of expanded clones in salivary glands was correlated with decreased salivary secretion and increased fibrosis. Although CD4+ cells are the majority of T cells within the glandular infiltrates of pSS patients, CD8+ T cells are also present. A percentage of these CD8+ T cells show an activated phenotype, as shown by a higher expression level of Human Leukocyte Antigen–DR isotype (HLA-DR, an MHC class II cell surface receptor). Increased proportions of HLA-DR^+^ T cells were associated with higher disease severity [26]. Additionally, in the blood of pSS patients with anti-SSA positivity, the increased frequencies of HLA-DR-expressing activated CD4+ and CD8+ T cells in blood was correlated with the EULAR Sjögren’s syndrome (SS) disease activity index (ESSDAI) scores [26]. Furthermore, the proportion of activated CD8+ T cells in blood was established by a multi-omic study based on whole blood transcriptomes, serum proteomes, and peripheral immunophenotyping, which identified pSS disease signatures dysregulated in widespread epigenomes, mRNAs, and proteins. [27]. For example, the expression of the chemokine receptor CXCR3 by activated CD8+ T cells in pSS patients may be important for their migration to the inflamed salivary glands and, as demonstrated in mice, the recruitment of activated CD8+ T cells to salivary gland tissue was dependent on CXCR3 [28]. We speculate that chronic antigen stimulation leading to systemic inflammation, reflected as higher ESSDAI scores, results in the activation of CD8+ T cells in secondary lymphoid organs, such as spleen, CXCR3 upregulation, and consequent migration to the salivary glands [29]. Whether CD8+ T cells, in turn, contribute to glandular dysfunction and fibrotic evolution or systemic disease activity is unknown (Figure 2).

#### 2.1.2. Autoimmune Treg Pro-Fibrotic Role

Recently, an intriguing role identified for a functional T cell subset named regulatory T lymphocytes (Treg) in tissue fibrosis has also begun to emerge [30]. Treg are crucial keepers of the immune system, shaping the development of fibrosis and causing lethal organ dysfunction [31]. Although some investigations have highlighted a controversial role for the Treg cells depending on the disease model, in recent years the majority of reports demonstrated an increase in the number of Treg in patients at the early phase of SSc [32]. Treg seem to be able to secrete transforming growth factor-β (TGF-β), the major pro-fibrotic factor, which induces myofibroblast activation and fibrosis [33]. In addition, the dysfunction of Treg cells in the early phase of SSc leads to autoimmunity and inflammation [34]. Notably, Treg cells have the capacity to differentiate in T-helper17 (Th17) cells under inflammatory conditions. Th17 cells secrete IL-17A, which could also promote myofibroblast transformation and fibrosis and was related to vasculopathy by promoting *endothelial inflammation*. A transcriptomic comparison between the early and late phases of SSc revealed a differentiated gene expression exclusively in Treg cells. Using an RNA-seq analysis to compare early SSc vs. late SSc patients, it was also reported that, in the early phase of SSc, enhancement of the oxidative phosphorylation pathway was observed which represents a metabolic sign of differentiation of Treg to Th17 cells [34]. Therefore, an imbalance between Treg and Th17 cells seems to be implicated in the pathogenesis of the early SSc. The contribution of Treg cells to the pathophysiology of SSc has been explained by several mechanisms, sometimes conflicting. In a normal function, Treg cells release inhibitory cytokines, such as IL-10, TGF-β, and IL-35, which function as immunosuppressive factors [35]. First, in SSc the suppressive effect of Treg cells is limited, causing an altered immune response and leading to chronic inflammation and fibrosis. The decreased inhibitory ability of Treg cells in SSc patients is attributed to the decreased production of TGF-β and IL-10 [36]. On the other hand, it is now established that the promotion of fibrosis by pro-fibrotic cytokines is produced by Treg cells. For example, TGF-β contributes to fibrotic pathology through the proliferation of fibroblasts, promoting collagen production and ECM secretion, and also induces the epithelial–mesenchymal transition (EMT). In addition, Treg cells seem to be able to differentiate into Th2-like cells in SSc and to promote fibrosis through the production of IL-4 and IL-13 [36]. By these mechanisms, Treg cells are thought to be associated with several aspects of immune dysregulation and fibrosis during SSc pathogenesis.

Recent findings, based on the study of the dysfunction and imbalance of Treg cells in pSS, have demonstrated a significantly lower frequency of Treg positive for pSTAT5 in pSS patients after IL-2 stimulation, compared with healthy controls [37]. No differences were demonstrated in other T-cell populations, indicating a specific impact of Tregs in pSS pathogenesis which, of course, will need to be clarified [37] (Figure 2). A decreased number of Treg cells was also demonstrated, specifically, in the patients with SLE, psoriatic arthritis, juvenile idiopathic arthritis, and autoimmune liver disease [31,38]. In the liver, a dual role of Tregs in fibrogenesis was detected because they are responsible for fibrosis promotion or immunosuppression [39]. In fact, a large number of Tregs are revealed in the fibrotic microenvironment in patients with hepatocellular carcinoma, in which it was observed that a reduction in Tregs promoted the regression of fibrosis [40]. Conversely, in autoimmune hepatitis, hepatic stellate cells (HSCs) were activated, whose function is to produce and accumulate ECM, a pivotal event in liver fibrosis. Simultaneously, HSCs selectively promote the survival and the activity of Tregs in an IL-2–dependent manner. Tregs can both protect HSCs from NK cell attack and, on the contrary, exert an inhibitory effect on HSCs, confirming the dual role of Tregs in liver fibrogenesis and the importance of equilibrium. The balance between Tregs which could convert to Th17 cells, seems, once again, fundamental in maintaining homoeostasis and immunoregulation; this mechanism, for reasons that are still unclear, can deregulate and leads to the production of pro-inflammatory cytokine by Th17, such as IL-17 and IL-22 [39].

#### 2.1.3. Emerging Pro-Fibrotic Role of T Follicular Helper Cells

T follicular helper (Tfh) cells have been identified as a distinct CD4+ helper T cell subset. They express a high level of surface markers, such as CXCR5, CD40L, inducible co-stimulator (ICOS), programmed cell death protein-1 (PD-1), and a downmodulation of C-C chemokine receptor type 7 (CCR7) [7,41]. Tfh cells are important modulators of B cell maturation and specialized to help B cells to produce high-affinity antibodies toward antigens and, thus, to develop an important humoral immune response [42,43]. Moreover, they are characterized by enhanced expression of IL-21 that promotes B cells’ differentiation into plasma cells and Ig isotype switching and by elevated production of the nuclear transcriptional repressor B cell lymphoma 6 (Bcl-6), essential for B cell function [43]. Many investigations have found severe proliferation and/or activation of Tfh cells in multiple autoimmune disorders characterized by intense fibrosis [7]. A possible role played by Tfh cells in the pathogenesis of SS was known; indeed, increased percentages of circulating Tfh cells (cTfh) have been demonstrated in peripheral blood [44] and in salivary glands of SS patients [45]. Several lines of evidence also support a pathogenic role of Tfh cells and IL-21 in human SLE. The Tfh surface marker ICOS seems to be crucial for optimal IL-21 production [46]. Higher plasma levels of IL-21 are found in SLE patients correlating with the number of switched memory B cells and with several markers of disease severity [43,47]. Findings from the literature are instead conflicting regarding Tfh cells’ frequencies in human RA. In some studies, augmented frequencies of cTfh cells in RA patients were observed, in particular in those with new-beginning disease [48]. On the role of Tfh in the fibrotic evolution of autoimmune diseases, few results are available in literature regarding the role of Tfh cells in SSc pathogenesis. A recent study provides evidence that Tfh cells induce skin fibrosis and correlate with dermal fibrosis [49] in SSc patients. Furthermore, it has been shown that the administration of both IL-21 and ICOS antibodies can effectively reduce skin fibrosis [50]. Moreover, an interesting recent report also evidenced that in patients with idiopathic pulmonary fibrosis (IPF), the levels of Tfh cells in the peripheral blood were increased [51]. Overall, from these data it can be deduced that Tfh cells may be involved in both immunological and fibrotic autoimmune disease, regulating autoreactive B cell expansion and fibroblast activation.

#### 2.1.4. Macrophages, Dendritic Cells, Mast Cells

In the complexity of the immune scenario, macrophages—key cells that classically initiate and sustain chronic inflammation in a simultaneous or parallel manner—are now recognized as capable of secreting fibrotic factors once activated [52]. Monocytes’/macrophages’ activation, due to the plasticity of these cells, could be an important step for the transition from the inflammatory to the fibrotic phase in SSc pathology. Through the release of fibro-proliferative factors, macrophages trigger the fibrotic process determining, for example, skin and lung SSc-related tissue fibrosis [52,53]. Consequently, an autocrine loop begins in which the release of fibrotic factors by macrophages drives the transformation of more monocytes/macrophages into cells with pro-fibrotic phenotype [52,53,54]. This cellular crosstalk occurs, clearly, in autoimmune hepatitis; hepatic resident macrophages have been shown to exert an intricate role in the initiation of inflammatory responses causing liver injury and can acquire a pro-fibrogenic phenotype that leads to aberrant tissue remodeling, culminating in liver and fibrosis and failure [55]. It is not possible to define exactly whether the macrophages involved in liver fibrosis belong to the M1 or M2 type. A switch between M1 and M2 phenotypes probably occurs because of their plasticity. M2 macrophages can be activated through IL-4Rα signaling, which determines liver inflammation and fibrosis. However, it has been demonstrated that the activation of M2-macrophages also represents a key event in viral-associated immune dysregulation and liver fibrosis [55].

Interestingly, in line with this concept, studies have highlighted that dendritic cells also display high plasticity after injury, driving pro-fibrotic inflammatory mechanisms in autoimmune diseases. Functional alterations of dendritic cells assist the immune processes favoring the altered T cell polarization and pro-fibrotic inflammation in the SSc [56]. DCs are commonly categorized into three major populations: the conventional DC (cDC)1, cDC2, and the plasmacytoid DC (pDC) [57]. pDCs are the subtype that appears to be more relevant for the development of fibrosis in SSc pathogenesis [56,58]. In patients with SSc, pDCs are mainly found in the skin and lungs [59], correlated with the severity of SSc disease [59]. Importantly, pDCs play a direct role in causing and maintaining fibrosis, as their depletion has been shown to improve skin and lung fibrosis. Furthermore, the presence of pDCs in the lungs appears to be a feature of pulmonary fibrosis, since their frequency in the lungs is similar in both SSc-interstitial lung disease (ILD) and idiopathic pulmonary fibrosis patients [60]. A key role in the pro-fibrotic activity of pDCs is done by CXCL4, secreted from pDCs of SSc patients, which creates an inflammatory environment in the tissues that they infiltrate. CXCL4 plays a central role in a feedback loop that contributes to increased inflammation and fibrosis [61]. It can directly promote the differentiation of different cell types into myofibroblasts, increasing the collagen and ECM component production and contributing to fibrosis [62]. Furthermore, increased levels of CXCL4 are found in the blood and skin of SSc patients [63], correlated with disease complications, such as ILD and pulmonary hypertension (PH). The production of CXCL4 from pDCs of SSc patients was also stimulated by TLR8, aberrantly expressed in this disease [64]. TLR8 induces the production of CXCL4 [64]. Additionally, TLR8 expression leads to an increased infiltration of pDCs into the tissues, exacerbating the disease and resulting in worse skin fibrosis [64].

**Figure 3:**
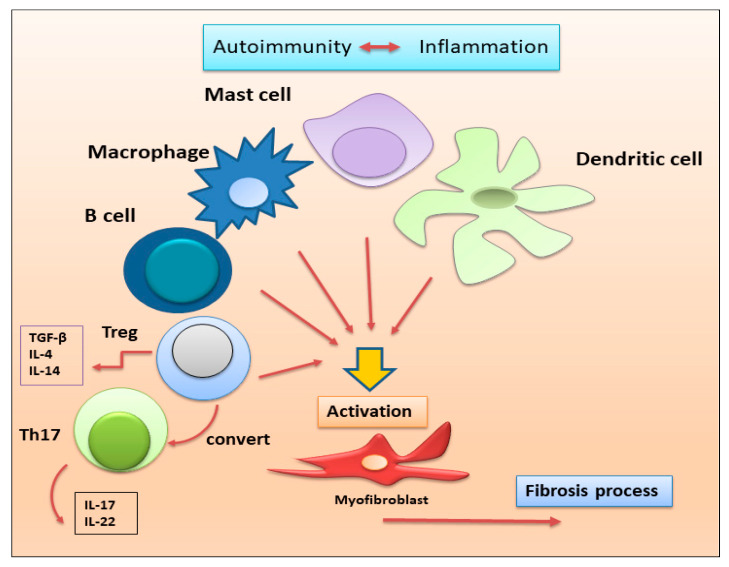
Immune cells recently linked to the fibrotic evolution of autoimmune diseases. In a condition of chronic inflammation, many immune cell populations with diverse functions are activated to produce multiple cytokines that lead to the proliferation and activation of myofibroblasts, directly involved in the development of fibrosis in various autoimmune diseases (Th17 = T helper 17; Treg = regulatory T lymphocytes).

Mast cells are immune cells mainly found in connective tissues with a well-established role in allergy and anaphylaxis. However, a great deal of evidence underlines their active role in tissue healing, angiogenesis, and exacerbation of chronic inflammation that characterizes autoimmune diseases [65]. Leehan and collaborators have recently investigated the role of mast cells in salivary gland fibrosis which is a pathological feature of pSS and positively correlates with high focus scores, but not with the age of the patients [66]. They demonstrated that mast cells are strongly associated with fibrosis and fatty infiltration of salivary glands that represent a biological response to gland injury. It is hypothesized that they promote fibrosis by interacting with local fibroblasts and producing enzymes responsible for cleavage and activation of metalloproteinases, which are important mediators of tissue injury and repair [66]. A schematic comprehensive overview of the involvement of immune cells in autoimmune-related fibrosis is reported in Figure 3.

### 2.2. Non-Immune Cells in Fibrotic Autoimmune Diseases

Recently, it has been proposed that also the non-immune cells, such as epithelial cells, endothelial cells, and fibroblasts may contribute to inflammation, autoimmunity, as well as fibrosis. Non-immune cells, when damaged or activated, release molecules involved in the regulation of several types of immune responses. Furthermore, the de novo production of bioactive factors by non-immune cells, in response to several stimuli, can influence immunological processes. Therefore, defects in the abilities of non-immune cells to mediate immune regulation may be involved in the pathogenicity of a series of inflammatory autoimmune diseases which often show a fibrotic organ evolution. This section will review the main non-immune cell types involved in fibrotic autoimmune diseases.

#### 2.2.1. Epithelial Cells

**Figure 4:**
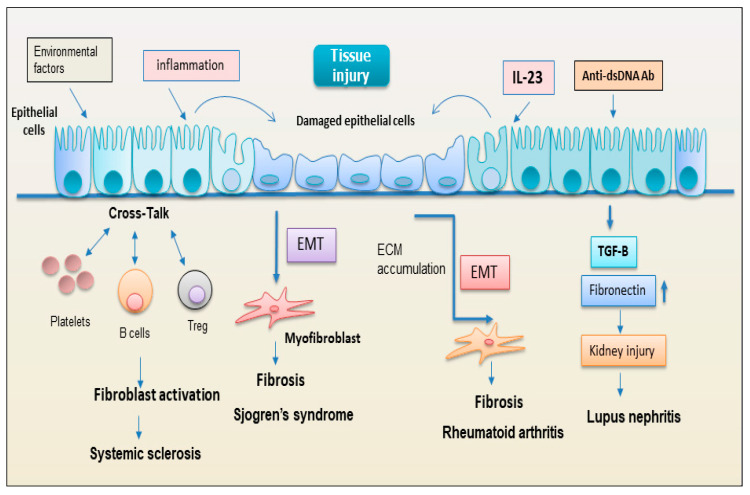
Representation of the hypothetical role of epithelial cells in the activation of the fibrotic program in various autoimmune diseases (anti-ds DNA Ab = anti-double-stranded DNA antibody; ECM = extracellular matrix; EMT = epithelial to mesenchymal transformation; TGF-β = tumor growth factor beta; Treg = regulatory T lymphocytes).

Epithelium includes various highly specialized cells that play critical roles in almost all biological processes, and they are considered essential to maintain tissue homeostasis in many organs. In this context, several studies have begun to examine the active role of epithelial cells in several autoimmune disorders characterized by fibrosis. The EMT program, under pathological conditions, can lead to the reduction of normal epithelial cells, destroying tissue architecture, inducing pathogenic activation of fibroblasts, and driving organ failure [8]. The knowledge of the molecular mechanisms that occur in the EMT program has demonstrated that the epithelial state of the cells initially considered immutable can undergo important changes in gene expression and post-translational regulation, leading to the repression of the epithelial characteristics and to the acquisition of mesenchymal characteristics displaying fibroblast-like morphology and cytoarchitecture [67]. Recently, considerable attention has been paid to chronic inflammatory disorders pSS in which the inflammatory status is often associated with pathological EMT-dependent salivary gland fibrosis [68]. Emerging evidence suggests that epithelial cells are also an important source of myofibroblasts in organ fibrosis [69], and this trans-differentiation is evaluated as a tightly specialized system of the EMT process that may be a central event in the salivary gland fibrosis [68]. The implications of these findings were very important and the recent explosion of knowledge in the biology of cellular differentiation has highlighted, for example, that differentiated cell type, such as a tubular or acinar salivary gland epithelial cell in pSS, with a wide set of glandular characteristics, such as secretion and transport, could radically change their transcriptional process, transcribing genes characteristic of the mesenchymal cell type [68,69,70,71]. Supporting this opinion, recent evidence highlights that salivary gland epithelial cells derived from healthy biopsies, when exposed to TGF-β1 stimulation, acquired a more fibroblast-like morphology [68,72,73]. Additionally, in SSc, recent studies have demonstrated anomalous phenotypes of the skin epithelium [74]. Indeed, phenotypically altered epithelial cells possibly explain the selective organ fibroses in the skin, oesophagus, and lung that occur in SSc [74]. In this context, several studies have begun to examine the functional role of tubular epithelial cells in the pathogenesis of lupus nephritis [75]. Renal tubular epithelial cells actively participate in the tubulointerstitial pathology of lupus nephritis through the expression of cytokines, chemokines, and pro-fibrotic factors, and play a crucial crosstalk with infiltrating cells of the immune system [75,76]. Findings suggest that anti-dsDNA antibodies that bind to the surface of renal tubular epithelial cells, but without cellular uptake and cytoplasmic/nuclear translocation, can promote tubule interstitial fibrosis and subsequently kidney dysfunction [77]. Yung et al. reported that anti-dsDNA antibodies derived from lupus nephritis patients induce a significant increase in the fibronectin expression in human renal tubular epithelial cells, a process dependent, in part, on the secretion of such fibrogenic factors as TGF-β [77]. These data suggest that fibrosis development in lupus nephritis is initiated and amplified via complex signaling pathways involving anti-dsDNA antibodies, fibronectin, and TGF-β in renal tubular epithelial cells [75]. A recent study has identified a key role for IL-23 as a pro-fibrotic molecule in RA-associated interstitial lung disease through the induction of the EMT-dependent transformation of somatic alveolar type I epithelial cells in fibroblast-like cells. The acquisition of a mesenchymal phenotype induced by IL-23 included increased deposition of ECM, the acquisition of invasiveness, and resistance to apoptosis—all events which may contribute to the formation of fibroblastic foci in fibrotic ILD, especially in the context of autoimmune pathology such as RA [78] (Figure 4).

#### 2.2.2. EMT: New Player Regulating the Interplay between the Immunity and Fibrosis

In recent years, epithelial to mesenchymal transition (EMT) has been extensively studied as a possible therapeutic target for fibrosis [72,73,79] and, therefore, a brief refresher in this area is needed. A better understanding of the crosstalk between chronic inflammation, autoimmunity, fibrosis, and EMT may represent an opportunity for the development of a broadly effective anti-fibrotic therapy in autoimmune diseases. Cells of multicellular organisms hire several phenotypes that have different functions, morphologies, and gene expression patterns, and, drastically, can undergo specific changes when subjected to determinants stimuli and microenvironments [80]. The inflammatory cells secrete crucial regulatory proteins, such as pro-fibrotic cytokines, chemokines, and growth factors, which can trigger the EMT process [81]. EMT is a highly dynamic process that often gives rise to a series of intermediate phenotypic states in which the cells progressively acquire mesenchymal markers without a concomitant complete loss of epithelial markers [82]. The expression of both mesenchymal and epithelial markers reflects the plasticity of cells depending on their environment [83]. Importantly, EMT leads to the early development of pathological organ fibrosis through paracrine signaling from the epithelium to potential fibroblasts [84]. The fibrotic process affects a variety of organs and tissues through the activation of specific molecular pathways [84]. However, two common hallmarks are evidenced: the critical role of the TGF-β and the implication of the inflammatory process, which are essential for initiating the fibrotic degeneration [72,79]. EMT is tightly related to fibrosis development in several organs and fibrogenesis represents the common response of organs and tissues to virtually all chronic repetitive injuries in multiple autoimmune disorders [72,73,79]. During chronic autoimmune diseases, inflammatory and epithelial cells produce fibrogenic mediators. In this context, TGF-β1 emerged as a crucial factor regulating interactions between epithelial and mesenchymal cells and fibroblasts proliferation [85]. One of the hallmarks of excessive pathological fibrogenesis is the acquisition by resident fibroblasts of a myofibroblasts contractile phenotype expressing high levels of *α-*Smooth muscle actin** (α-SMA). Additional immune cells are recruited into the fibrotic tissue, amplifying the fibrotic response by the secretion of chemokines, cytokines, and growth factors responsible for the differentiation of other myofibroblasts implicated in ECM deposition [86]. The principal EMT pathway is mediated by Smad and we can indicate it as TGF-β1/SMAD/Snail pathway; it is a particularly interesting system active in the EMT-dependent fibrotic process in a number of diseases [72,87]. Alternatively, or parallel to Smads pathways, TGF-β1 also utilizes a multitude of intracellular non-canonical, non-Smads TGF-β-mediated cascade triggered by the binding of ligands different from TGF-β family members to tyrosine kinase receptors [88,89]. This may suggest that the therapeutic use of TGF-β signaling inhibitors, actually used in cancer, may also be hypothetically extended to the treatment of inflammatory autoimmune disorders, but future investigations are needed to prove this hypothesis.

#### 2.2.3. Endothelial Cell

**Figure 5:**
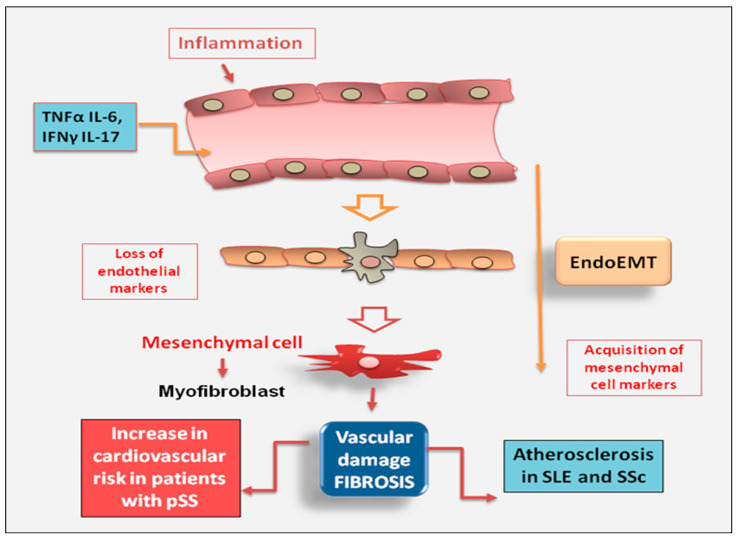
In chronic autoimmune diseases, endothelial cells are often the primary site of inflammation that triggers the downstream molecular events of fibrosis. The activation of myofibroblasts in various autoimmune diseases such as SLE (Systemic Lupus Erythematosus) and SSc (systemic sclerosis) may result from the phenotypic conversion of endothelial cells into activated mesenchymal cells, a process known as endothelial to mesenchymal transition (EndoMT) (IFN-γ = interferon gamma; pSS = primary Sjögren’s syndrome; TNF-α = tumor necrosis factor-alpha).

Dysregulation of endothelial cell function is proposed as a crucial start event, leading to vascular remodeling linked to fibroproliferative vasculopathy. Impaired angiogenesis may be induced by the massive proliferation of fibroblasts observed in some autoimmune diseases characterized by intense pathological fibrosis. New insights have evidenced that myofibroblasts involved in tissue fibrosis can still derive from endothelial cells through a process known as EndoMT [90,91]. It is a non-malignant phenomenon of cellular trans-differentiation in which endothelial cells undergo a phenotypical change where they lose vascular epithelial factors and acquire mesenchymal cell markers [92]. Among systemic autoimmune diseases, endothelial dysfunction has been extensively studied in SLE. In SLE patients, endothelial dysfunction is the main factor of vascular aging and pre-clinical atherosclerosis that leads to vascular fibrosis, contributing to the early onset of cardiovascular disease and cardiovascular mortality [93]. Interesting studies have highlighted that endothelial dysfunction also occurs in patients with pSS. Recent epidemiologic data indicate an increase in cardiovascular risk in patients with pSS and endothelial dysregulation may cause vascular fibrosis, leading to arterial stiffness, which precedes the development of high blood pressure [94]. This study demonstrates that patients with pSS, without clinically evident cardiovascular disease or without concomitant cardiovascular risk factors, have an altered endothelial function and a massive proliferation of fibroblasts, which suggest a higher susceptibility to the development of vascular fibrosis [94]. Therefore, induction of pro-inflammatory cytokines, such as TNFα and IL-6, involved in atherosclerotic damage, in combination with IFNγ and IL-17, reduces the number of smooth muscle cells, increases collagen production, and favors fibrosis development, with subsequent formation of fibrous atherosclerotic plaque [95]. In this intriguing scenario, the evidence that circulating biomarkers of inflammation predict future cardiovascular events in patients with pSS further reinforces the strict interplay between chronic inflammation and atherosclerosis. Furthermore, subclinical cardiovascular involvement is directly related to elevated inflammatory injury, postulating that inflammation and disease activity are cardiovascular disease risk factors in patients with pSS [94]. In the case of SSc, recent reports have evidenced that this disease was characterized by a massive accumulation of fibroblasts and myofibroblasts and by an abnormal production of interstitial collagens and extracellular matrix components, and was identified by the dysregulation of endothelial cell activity as a pivotal event that contribute to vasculopathy in SSc [92,96] (Figure 5).

#### 2.2.4. Fibroblasts

Traditionally, fibroblasts were considered the main contributory cells to the structural integrity of tissues; only recently have they been recognized as cells that exhibit a dynamic role in physiological or pathological processes [97,98] and are considered active producers of inflammatory cytokines and chemokines. An emerging concept, derived from experimental research on fibroblasts in inflammatory and fibrotic diseases, is that their differentiation is maintained by extrinsic and intrinsic danger signals and local microenvironment-derived morphogens [99,100]. Moreover, fibroblasts can initiate the early molecular processes leading to inflammatory events [100] and, consequently, can be involved with a prominent role in the pathogenesis of fibrotic autoimmune conditions [100].

An interesting paper by Wang, W. et al. [101] demonstrated an incisive role of fibroblasts in systemic sclerosis. In this study, fibroblasts isolated from skin and lung biopsies of patients with systemic sclerosis was analyzed and an altered expression of the A20 gene was detected; A20 is a gene strongly linked with disease susceptibility and fibrotic manifestations [101]. According to some reports it was demonstrated that A20 expression in fibroblasts can inhibit the fibrotic process, whereas its negative transcriptional regulator, called DREAM (downstream regulatory element antagonist modulator), promotes fibrotic processes [102]. The authors proposed that the upregulation of DREAM in systemic sclerosis fibroblasts underlies suppression of A20, which in turn contributes to unchecked pro-fibrotic signaling in stimulated fibroblasts [101]. Interestingly, targeting the A20–DREAM regulatory network could represent a novel therapeutic approach in systemic sclerosis [102].

New reports have documented that the immunomodulatory role of the fibroblasts derived from salivary glands was discovered in a primary site affected by the pSS [103]. Interestingly, these specific clusters of fibroblasts constitute the formation of tertiary lymphoid structures, which are linked to severe disease and can determine a risk factor for the development of lymphoma in pSS [103]. Recent advances in single-cell profiling techniques have demonstrated the presence of fibroblasts in inflamed salivary glands tissue, providing evidence of the existence of inflammation-associated fibroblasts in chronically inflamed tissues [104]. Clusters of fibroblasts were identified as key players in the development of renal fibrosis and, in particular, in lupus nephritis [105].

New discoveries highlighted as myofibroblasts are the main actors involved in renal fibrogenesis. Interestingly, the differentiation of fibroblasts to myofibroblasts is a key cellular event in many autoimmune fibrotic disorders [106].

Single-cell sequencing has demonstrated that myofibroblasts have different gene expression profiles with dynamic changes in fibrosis of different organs [107]. Myofibroblasts, armed with myosin and smooth muscle actin (α-SMA), become able to secrete TGFβ, VEGF, CTGF, IL-1, IL-6, and IL-8 [108].

**Figure 6:**
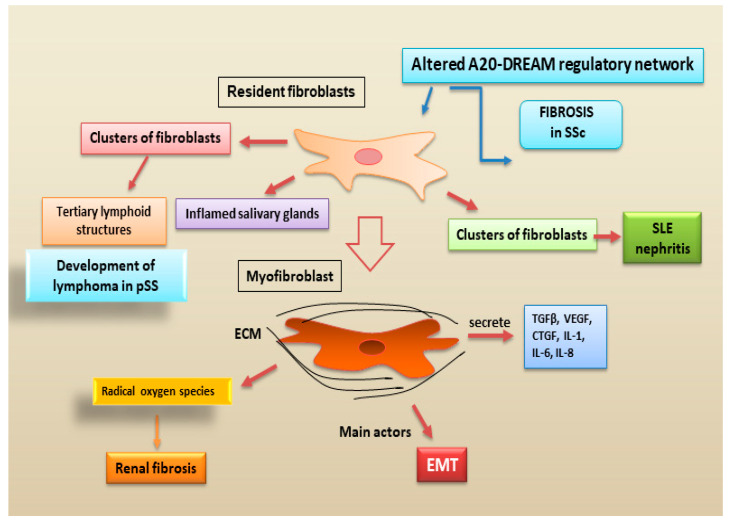
Recent advances in the pro-fibrotic role of fibroblasts in autoimmune diseases. The traditional view that fibroblasts represent purely structural elements has been gradually replaced by the acknowledgement that they are dynamic cells actively involved in the evolution from inflammatory states to fibrosis (A20-DREAM = A20-downstream regulatory element antagonist modulator; CTGF = connective tissue growth factor; ECM = extracellular matrix; EMT = epithelial to mesenchymal transition; Il: interleukin; pSS = primary Sjögren’s syndrome; SLE = Systemic Lupus Erythematosus; SSc = systemic sclerosis; TGF-β = tumor growth factor beta; VEGF = vascular endothelial growth factor).

It has been suggested that myofibroblasts localized in renal fibrotic tissue may derive from different precursor resident cells, including fibroblasts and epithelial cells [109]. Moreover, myofibroblasts not only contribute to deposition of ECM, but they can produce radical oxygen species and, through their intrinsic contractile properties, can alter renal tissue architecture [109]. Their pathogenic role in renal fibrosis has been discovered in different murine models in which the removal of myofibroblasts can reduce fibrogenesis [98]. Moreover, myofibroblasts are considered as one of the principal participants in the final point of EMT. After an acute insult, a temporary activation of the EMT process is considered of fundamental importance in renal repair [105,110]. The identification of key morphogen signals that regulate fibroblast differentiation could provide a therapeutic opportunity (Figure 6).

## 3. Conclusions

The fibrotic consequences of various primary autoimmune diseases, characterized by tissue damage resulting from chronic inflammatory conditions, remain a major unsolved diagnostic and therapeutic challenge. From experimental experience, it seems that all fibrotic tissues derived from autoimmune patients display signs of chronic immunologically mediated inflammation during the earliest periods of fibrosis. In these initial stages of fibrotic evolution, a predominant role is certainly played by immune cells, although some questions remain open about the specificity of lymphocyte subtypes occurring in fibrotic tissue, as well as about a possible imbalance of pro- and anti-fibrotic factors produced by components of the immune cells infiltrate. For example, the precise mechanism underlying the immune reaction in fibrogenesis mediated by Tregs, probably depending on different immune microenvironments and molecular pathways, is still unclear and will require further investigation. Actually, there is accumulating evidence showing that non-immune cells, such as epithelial cells, endothelial cells, and fibroblasts are cells with important immunomodulatory properties, play a pivotal role in the switch to chronic inflammation. Determining the exact contribution of these mechanisms remains a challenge, as they are at the cross-point of multiple regulatory networks also involving immune and non-immune cells and this, in an autoimmune condition in which the immune system works in an altered way, makes the scenario even more complex. For example, whether EMT activation may interfere with the crosstalk between epithelial cells, mesenchymal cells, and immune cells, stimulating fibrotic evolution, remains elusive. Since valid biomarkers for the diagnosis and staging of autoimmune-related fibrosis are not yet available, more detailed knowledge on the cellular and molecular basis of fibrogenesis is urgently needed. From this point of view, a better knowledge of the non-immune cells contribution to autoimmune fibrosis should help to appreciate the reasons underlying the actual clinical failures and design more effective therapies.

## Figures and Tables

**Table 1:** Autoantigens associated with autoimmune fibrosis. (RF = rheumatoid factor; RNP = ribonucleoprotein).

Antigen Location	Antigen	Fibrosis Autoimmune Diseases
**Nuclear**	Ro-RNP complex	Systemic lupus erythematosus, Sjögren’s syndrome
	La antigen	Systemic lupus erythematosus, Sjögren’s syndrome
	Small nuclear RNP	Systemic lupus erythematosus, Idiopathic pulmonary fibrosis
	Chromatin	Autoimmune hepatitis, Systemic sclerosis
	dsDNA	Systemic lupus erythematosus, Autoimmune hepatitis
	Topoisomerase I	Systemic sclerosis
	Centromere	Systemic sclerosis
**Modified proteins**	Citrullinated proteins	Rheumatoid arthritis, Idiopathic pulmonary fibrosis
	Carbamylated proteins	Rheumatoid arthritis
**Extracellular**	RF (IgG)	Rheumatoid arthritis

## Data Availability

Not applicable.
